# Supplementary figures and images for: Lineage space and the propensity of bacterial cells to undergo growth transitions
Source: PLoS Comput Biol. 2018 Aug 22;14(8):e1006380. doi: 10.1371/journal.pcbi.1006380 (PMC6122811; doi:10.1371/journal.pcbi.1006380)

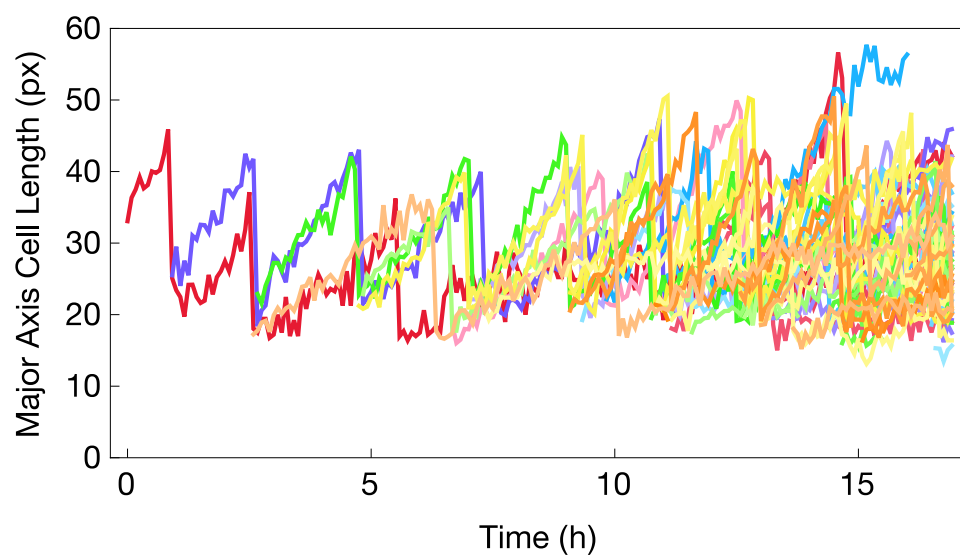

Supplement: S1 Fig — (PDF) [file pcbi.1006380.s003.pdf]

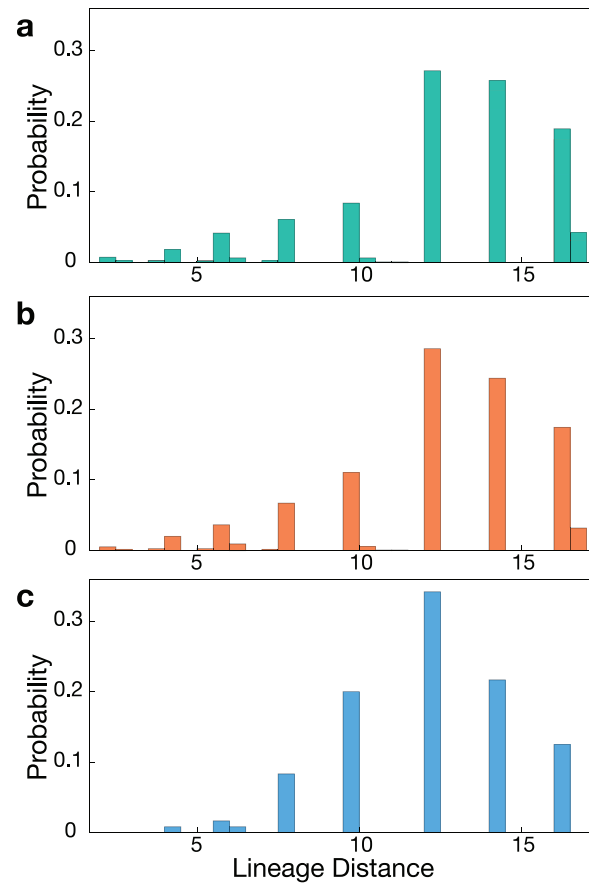

Supplement: S2 Fig — All cells (a), only non-growth-arrested cells (b), and only growth-arrested cells (c) in the lineage shown in Fig 2D. p < 0.01 for growth-arrested cells to not to have lower lineage distances versus either of the other two groups (one-tailed Mann-Whitney U test). (PDF) [file pcbi.1006380.s004.pdf]

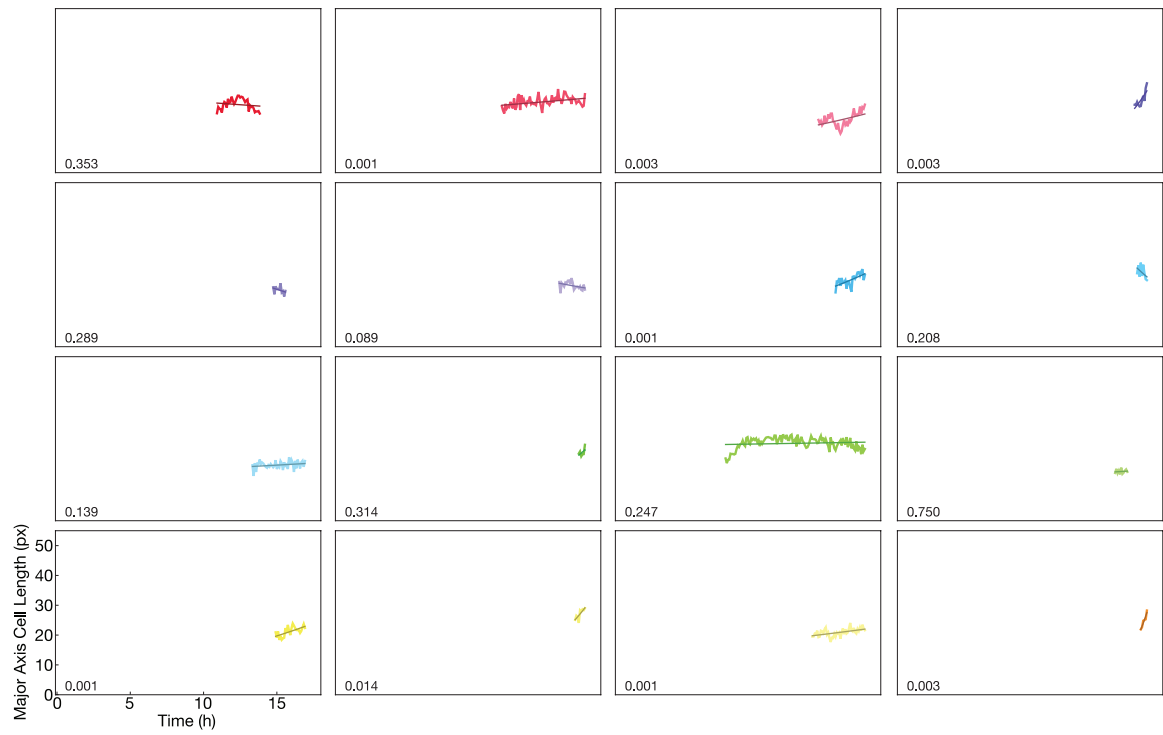

Supplement: S3 Fig — Lengths of growth arrest-prone cells between divisions were tested for a significant fit to an exponential growth model in the growth arrest-prone condition. These cases failed the significance test with a Bonferroni-adjusted α = 0.05 (adjusted value = 0.000424). (PDF) [file pcbi.1006380.s005.pdf]

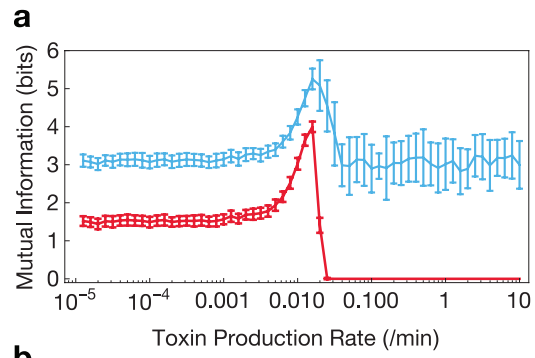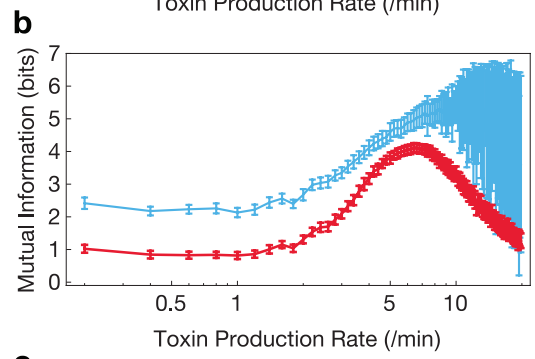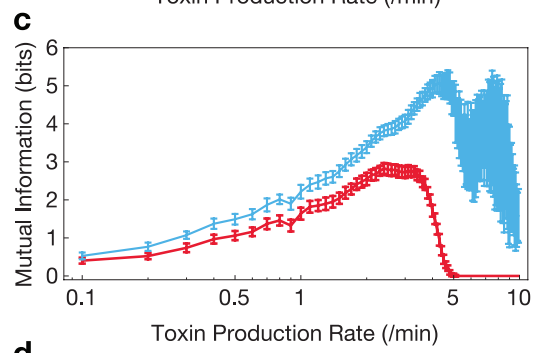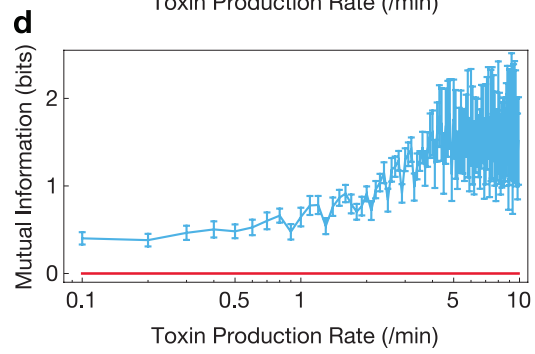

Supplement: S4 Fig — a. Altering toxin degradation rates to represent the precise mechanism of toxin-antitoxin systems. b. Altering toxin and antitoxin production so that they are bursty with a telegraph (ON-OFF) model. c. Increasing toxicity with parameter α = 0.3. d. Eliminating growth feedback (α = 0) eliminates the peak of mutual information along with the lack of macroscopic growth regulation. (PDF) [file pcbi.1006380.s006.pdf]
